# Chronic Duodenal Indigestion in Children

**Published:** 1917-02

**Authors:** John Foote

**Affiliations:** Washington, D. C.


### Chronic Duodenal Indigestion in Children.

BY JOHN FOOTE, M. D., WASHINGTON, D. C.

This condition is said to occur most frequently in children after the first year, and especially in those who have suffered from dietetic errors, usually with antecedent contagious diseases, or from prolonged intestinal infections, and this is fully covered by Foote in the December *International Clinics.* This form of indigestion seems to be accompanied by deficiency or pancreatic ferments, especially lipase. A mild duodenitis, which either passes up the pancreatic duct, or diminished hormone formation, seems responsible for the condition.

Diminished bile production may also be a factor. Anemia, loss of weight and mental underdevelopment occur. Large pendulous abdomen are common. Bottle feeding has been employed. Fever may be encountered, vomiting almost never. The number of daily stools varies from three to twelve. They are thin, contain some mucus and flakes of whitish material and have a very foul odor. They give an acid reaction and microscopically contain not only large quantities of fat soaps, but also a considerable amount of neutral fat but rarely starch granules. It is to be differentiated from mesenteric tuberculosis and acute duodenal indigestion. The treatment consists in reducing the food elements which have proven indigestible, namely, the fat, and stimulating enzyme production by the administration of hydrochloric acid and pancreatic ferments.—*International Clinics.*

---

Dr. W. G. Emory, of Bryan, Texas, died February 13 from wounds received when he was shot by L. D. Webster, of Austin.

